# Is France Once Again Looking for a Scapegoat?

**DOI:** 10.20411/pai.v6i2.490

**Published:** 2021-12-29

**Authors:** Michael M. Lederman, Jeffrey S. Flier, Peter Hale, Ashley T. Haase, William Powderly, Peter Reiss, Guido Silvestri, Rafick P. Sekaly, Mirko Paiardini, Drew Weissman, Daniel R. Kuritzkes, Leonard H. Calabrese, Peter Agre, Gustavo Reyes-Teran, Alan L. Landay, Sharon Lewin, Douglas D. Richman, Paul Volberding, Peter W. Hunt, Mauro Schechter

**Affiliations:** 1 Case Western Reserve University School of Medicine, University Hospitals Case Medical Center, Cleveland, Ohio; 2 Harvard Medical School, Boston, Massachusetts; 3 Foundation for Vaccine Research, Washington DC; 4 University of Minnesota, Minneapolis, Minnesota; 5 Washington University, St. Louis, Missouri; 6 University of Amsterdam, The Netherlands; 7 Emory University, Yerkes Primate Center, Atlanta, Georgia; 8 Perelman School of Medicine, University of Pennsylvania, Philadelphia, Pennsylvania; 9 Brigham and Women's Hospital, Harvard Medical School, Boston, Massachusetts; 10 Cleveland Clinic, Lerner College of Medicine, Case Western Reserve University, Cleveland, Ohio; 11 Bloomberg School of Public Health, Johns Hopkins University, Baltimore, Maryland; 12 National Institutes of Health, Mexico City, Mexico; 13 Rush University, Chicago, Illinois; 14 The Peter Doherty Institute for Infection and Immunity, University of Melbourne, Melbourne Australia; 15 University of California San Diego, San Diego, California; 16 University of California San Francisco, San Francisco, California; 17 Universidade Federal do Rio De Janeiro, Brazil

**Keywords:** COVID-19, pandemic response, public health, epidemiology, Cour de Justice de la Republique, PCR, facemasks, inquest

## Abstract

On September 10, 2021, a special tribunal established by the French government launched an inquiry into the activities of former health minister Dr. Agnes Buzyn who was charged with “endangering the lives of others”. It is surprising to learn of this accusation and inquiry into the actions of a public health official whose response to the epidemic was, to all appearances, exemplary.

On September 10, 2021, a special tribunal established by the French government to investigate ministerial misconduct (Cour de Justice de la Republique – CJR) launched an inquiry into the activities of former health minister Agnes Buzyn who is being charged with “endangering the lives of others” [1, 2] Other members of the French government also may be charged. This inquiry is expected to last years. Le Journal du Dimanche reports that this reflects her being held responsible for the government's failure to find her replacement for 6 months after her departure and because early in the epidemic, masks were not broadly available to the general public [3]. We focus this commentary on Dr. Buzyn since she is the first to be charged, she served as Minister of Health for only 6 weeks after COVID-19 was first identified and because this episode has ugly echoes of the Dreyfus affair, a political scandal that rocked France from 1894 until 1906 when a Jewish military officer was wrongly accused of betraying military secrets. It is difficult to understand why such an investigation is taking place when to all appearances Dr. Buzyn's response to the emerging pandemic was exemplary. Her health alert responses to news of this infection in Wuhan and her rapid calls for action antedated those of other European and American health authorities. The following chronicles the actions she took during those first 6 weeks and reflects her testimony to the French Senate.

On January 2, 2020, one day after WHO reported 27 instances of pneumonia in Wuhan, Buzyn declared a Level 1 alert for the French Center for Health Crisis. On January 14, one day after the first case outside China was reported (in Thailand) Buzyn's Ministry of Health notified all health professionals, hospitals, and nursing homes. On January 21, after the European CDC stated that risk of importation into the EU was low, Buzyn restated and modified this saying “importation cannot be excluded as there are flights between France and Wuhan”. She immediately started daily press briefings for the public. As unofficial communications suggested the possibility of human-to-human transmission, she was the first to inquire about the availability of masks, and within 3 days ordered millions more for health care workers.

On January 24, the first 3 cases of COVID-19 (from Wuhan) were detected in France; Buzyn called for contacts to be traced and quarantined and sent a third health alert to all health professionals. Buzyn called the European Health Commissioner requesting a meeting of all ministers of health and called the WHO Director General Dr. Tedros to understand why WHO was not declaring an international public health emergency.

The next day, the European CDC congratulated France on its preparedness. Buzyn ordered her staff to collect data on ICU capacity, ventilators, and protective equipment stocks. She called other EU health ministers and learned on January 26 that only 3 of 27 EU nations considered that a special meeting of all ministers was warranted. Buzyn brought representatives of French hospitals and health professionals together on January 27 raising the crisis level to 3 providing for mandatory quarantine of contacts.

The same day the Pasteur Institute announced that its diagnostic PCR test was ready for deployment in all hospitals in France. Although the United States FDA approved some PCR tests on February 29th, it was not until March 12, 2020, that all 50 US states were able to perform diagnostic PCR tests for COVID. In Holland, testing capacity was low until June 1, 2020 [4]

On January 28, Buzyn's Ministry of Health issued its fourth health alert to physicians and health care facilities. As of January 29, there had been 5 cases in France, all imported. Contacts were traced and quarantined. On January 30, Buzyn met with the research community to prepare for clinical trials if and when the epidemic would reach France. WHO declared an international public health emergency but recommended against closing borders. Nonetheless, Air France ended flights to China on February 2.

On January 31, Buzyn commandeered all public health residents and interns to help regional agencies trace contacts in case of outbreaks. The European CDC stated that human-to-human transmission risk in the EU was low if early detection, contact tracing, and quarantine practices were followed but high if detection was late. On February 7, the Ministry of Health placed orders to restock personal protective equipment, and the first cluster of 5 cases was found in France. Following Buzyn's instructions, contacts were traced and quarantined.

On February 16, Buzyn resigned at the request of President Macron to stand for election as Mayor of Paris. By that date there had been 12 recognized cases of COVID in France. Outbreaks of COVID were first detected in France on February 25. WHO did not declare a pandemic until March 11.

Buzyn has served with honor and distinction as one of the most competent and respected health ministers in France between May 2017 and February 16, 2020.

It's hard to know why France is so eager to find a scapegoat. There are a number of possibilities that include political animus against a Macron appointee, a settling of old political scores, the resentment of a powerful woman, or the dangerous and growing distrust and anger directed against scientists during this pandemic where public health officials and their families have been threatened by members of the public [2, 5]. As Dreyfus was, Buzyn has also been the target of anti-Semitism. One social media cartoon showed her dropping poison into wells [6]. Crises like pandemics offer to some, opportunities for attacks on old and new enemies by those with axes to grind. Dr. Buzyn deserves better than this for her service.

As a republic that has contributed so much to science and stands as a birthplace of democracy, France also deserves better. As scientists, we must ask the CJR for an unbiased resolution of these accusations. Based on available evidence we anticipate her full exoneration and hope she receives an apology by the authorities for judiciary overreach.
